# Use of once-daily raltegravir-based HAART in HIV-infected injection drug users

**DOI:** 10.1186/1758-2652-13-S4-P36

**Published:** 2010-11-08

**Authors:** HK Tossonian, JD Raffa, O Alenezi, K Anderson, S Jassemi, K Wight, S DeVlaming, B Conway

**Affiliations:** 1 University of British Columbia, Vancouver, Canada;; 2 University of Waterloo, Waterloo, Canada;; 3 Pender Community Health Centre, Vancouver Coastal Health, Vancouver, Canada

## Purpose

Within a prospective observational study, we measured adjustment of methadone doses and responses to treatment after once-daily raltegravir (RGV)-based highly active antiretroviral therapy (HAART) was initiated in injection drug users (IDUs).

## Methods

We evaluated HIV-infected IDUs attending an inner city clinic in Vancouver who were receiving RGV-based HAART and methadone within a directly observed therapy program. Follow-up was according to clinical standards, with changes in methadone dose being made as required to achieve clinical stabilization within the first month of HAART. The change in methadone dosing associated with the initiation of HAART was calculated as the difference between the post- and pre-HAART methadone doses. The most recent on treatment CD4 cell count and HIV plasma viral load were used to evaluate HAART efficacy after initiation of therapy.

## Results

The study included 34 subjects (9 female) with a median follow-up period of 16.5 months. All patients were treatment experienced and co-infected with hepatitis C virus. Most patients received RGV-based HAART along with emtricitabine and tenofovir (n=16) or lamivudine and abacavir (n=9). At baseline, the mean methadone dose, mean CD4 cell count and median plasma viral load were 97.4 mg/day, 286 cells/mm^3^ and 243 copies/mL, respectively. At month 3, the mean methadone dose was 97.9 mg/day with the observed mean methadone dose change from baseline being 0.4 mg/day (p=NS). In these patients, 7 (21%) required increases, 8 (24%) required decreases, while 19 (56%) required no change in daily methadone dose from baseline. At most recent follow-up, the mean CD4 cell count was 355 cells/mm^3^ while virologic suppression (HIV RNA <50 and <400 copies/mL) was achieved in 23 (68%) and 31 (91%) of patients receiving RGV-based therapy.

## Conclusions

Lack of drug interactions with methadone and improved immunologic and virologic responses support the use of once-daily RGV-based HAART in this vulnerable population.

